# Cation-Exchange in Metal-Organic Framework as a Strategy to Obtain New Material for Ascorbic Acid Detection

**DOI:** 10.3390/nano12244480

**Published:** 2022-12-18

**Authors:** Weronika Bodylska, Marzena Fandzloch, Rafał Szukiewicz, Anna Lukowiak

**Affiliations:** 1 Institute of Low Temperature and Structure Research, Polish Academy of Sciences, Okólna 2, 50-422 Wrocław, Poland; 2 Faculty of Physics and Astronomy, University of Wrocław, pl. M. Borna 9, 50-204 Wrocław, Poland

**Keywords:** metal–organic framework, ascorbic acid, detection, europium, photoluminescence

## Abstract

Ascorbic acid (AA) is an important biomolecule, the deficiency or maladjustment of which is associated with the symptoms of many diseases (e.g., cardiovascular disease or cancer). Therefore, there is a need to develop a fluorescent probe capable of detecting AA in aqueous media. Here, we report the synthesis, structural, and spectroscopic characterization (by means of, e.g., XRD, XPS, IR and Raman spectroscopy, TG, SEM, and EDS analyses), as well as the photoluminescent properties of a metal–organic framework (MOF) based on Cu^2+^ and Eu^3+^ ions. The ion-exchange process of the extraframework cation in anionic Cu-based MOF is proposed as an appropriate strategy to obtain a new material with a nondisturbed structure and a sensitivity to interaction with AA. Accordingly, a novel Eu[Cu_3_(μ_3_-OH)(μ_3_-4-carboxypyrazolato)_3_] compound for the selective optical detection of AA with a short detection time of 5 min is described.

## 1. Introduction

The detection of ascorbic acid (AA) is crucial for human health; hence, it plays an integral role in the physiological functioning of the body [1]. It not only has an antioxidant effect but also acts as a neuromodulator. Varying levels of AA in biological fluids may be used for the measurement of oxidative stress levels in the body’s metabolism. An increased level of oxidative stress has been linked to diabetes, cancer, hepatic and neurodegenerative diseases, and mental disorders [2], thus it is important to monitor its level. 

To this day, various techniques, including electrophoresis [3], chemiluminescence [4], titrimetry [5], fluorescence [6,7,8,9], and electrochemical techniques [10,11,12] have been reported to detect AA. Two of the most used methods are fluorescence and electrochemical measurements. Electrochemical sensors for AA detection may be classified into two types: enzymatic and nonenzymatic [12]. The difference between them is that the work of enzymatic sensors is based on the catalytic efficiency of enzymes, while nonenzymatic sensors utilize the catalytic properties of chemical components. Considering that AA may be easily electrochemically oxidized in aqueous solutions, its electrochemical detection is a valid, fast, and simple, as well as sensitive and selective method [11], but it also has some drawbacks. In the case of enzymatic sensors, enzyme denaturation and leaching out of the electrode surface may lead to poor stability. What is more, the temperature and humidity may also affect the sensing using biological electrodes. It may be omitted by direct electrooxidation using nanomaterials. However, these nonenzymatic sensors have other disadvantages, such as a poor selectivity and narrow detection range [12]. Optical sensing using fluorescence has also been a popular option for AA sensing due to the high sensitivity and low detection limit. Moreover, optical sensors are usually chemically inert and capable of monitoring a wide range of chemical parameters [13]. Various types of materials may be used for AA optical detection, such as, e.g., quantum dots [6], silver nanoparticles [7,8], rare-earth-doped optical fibers [9], or metal–organic frameworks (MOFs) [14].

MOFs, also known as porous coordination polymers, are a class of functional materials. Due to the possibility of adjusting chemical and physical features, MOF investigations have become one of the most rapidly growing areas in chemistry and materials science [15,16]. MOFs have various advantages over typical porous materials, such as a diverse composition and micro-/nanoscale structures [17], a large specific surface area [18], or a high porosity [19]. MOFs’ structural properties may be freely tailored; thus, they can be applied in various fields, including gas storage and separation [20], drug delivery [21,22,23], catalysis [24,25] or electrocatalysis [26], water purification [27], or even for micro-supercapacitors [28].

Recently, MOFs based on lanthanides have become an interesting field of research and have been widely used for optical (bio)sensing. The luminescent properties of MOFs are sensitive and depend on their structure, the coordination environment of the metal ions, pore surface, and the interaction with guest species [29]. MOFs exhibit certain advantages over conventional luminescent sensors, such as their constant porosity for the reversible uptake of sensing substrates and their adjustable pore size for the selective detection of small molecules and ions. In addition, the presence of free basic or acid Lewis sites and open metal sites in MOFs can cause various types of interactions with small molecules [29]. What is more, the structural regularities of MOFs may improve sensing performance. They exhibit sharp emission lines, large Stokes shifts, and a high quantum yield. In addition, the luminescence of MOFs may arise from metal ions, ligands, and guest molecules, which enables the use of ratiometric sensors through the design of multiemission materials. Considering the mentioned advantages, MOF-based luminescent sensors have been recently used for the detection of ions, explosives, volatile organic compounds, and biomolecules [30].

One of the most commonly used rare-earth elements for MOF-based sensors is europium, due to its bright emission and its detectable intensity changes. Eu^3+^ emits a red light with emission bands at around 590, 614, 651, and 697 nm, which are assigned to the ^5^D_0_→^7^F_n_ (*n* = 1–4) transitions. The luminescent properties of Eu^3+^ ions may be adjusted by the guest molecule by blocking the excitation light, regulating the energy transfer process, or quenching [31]. Eu-based MOFs may be used for selective sensing of many analytes such as copper [31], iron [32], or arsenate ions [33], antibiotics [34], dyes [35], biological markers [36], or cofactors such as ascorbic acid (AA) [37,38]. 

It is worth noting that designing a heterometallic–organic framework consisting of lanthanides and transition metals enables one to combine the merits of both ions. Both transition- and lanthanide-MOFs have, among others, remarkable optical and catalytic properties [39,40,41,42]. Among such structures, a heterometallic–organic framework based on europium and copper ions for AA detection was proposed by Zheng et al. [37]. The novel MOF structure [EuCu(pydc)_2_(ox)_0.5_(H_2_O)_3_∙1.5H_2_O]_2*n*_ selectively detected AA in a wide concentration range (55 nM to +∞) by introducing H_2_O_2_ to the system and recording the variation of luminescence intensity. Moreover, it provided a high-efficiency detection in human serum samples, which is promising for applications in medical diagnosis.

Following this topic, this study aimed to obtain new material for selective AA detection based on Eu^3+^ and Cu-MOF. As an effective synthesis strategy, the ion-exchange processes of the extraframework cation [43] in a unique anionic MOF NH_4_[Cu_3_(μ_3_-OH)(μ_3_-4-carboxypyrazolato)_3_] (NH_4_@Cu-MOF) is proposed. The report includes information on the synthesis, structure, thermal stability, as well as changes in textural and luminescent properties of the system (Eu@Cu-MOF) after interaction with AA.

## 2. Experimental 

### 2.1. Materials

All chemicals were commercially available and used without further purification. Ethanol (96%), ammonia hydroxide (25%), glucose, arginine, citric acid (99.4%), hydrochloric acid (35–38%), and ascorbic acid were purchased from Avantor Performance Materials Poland S.A. Europium(III) nitrate hexahydrate (99%), formic acid (>96%), and 1H-Pyrazole-4-carboxylic acid were purchased from Thermo Fisher Scientific. Copper(II) nitrate hexahydrate, leucine, and glutamic acid were purchased from Sigma Aldrich (St. Louis, MO, USA).

### 2.2. Synthesis of Eu@Cu-MOF

**Figure 1:**
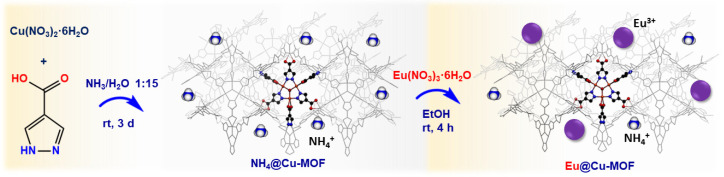
Synthesis scheme of Eu@Cu-MOF.

The new system was obtained in a two-stage procedure (Figure 1). In the first step, NH_4_@Cu-MOF was prepared according to the synthesis scheme previously described by the Navarro group [43]. For this purpose, 1H-pyrazole-4-carboxylic acid (2 mmol) and copper(II) nitrate hexahydrate (2 mmol) were dissolved in an aqueous ammonia solution (NH_4_OH/H_2_O = 1:15, 30 mL) and left for 3 days while dark blue crystals formed. Afterward, these crystals were centrifuged, washed with distilled water (2 × 20 mL) and ethanol (1 × 20 mL), and dried for 24 h at 50 °C. In the second step, to the aqueous europium nitrate solution (0.01 M, 6 mL), 50 mg of NH_4_@Cu-MOF was added and mixed for 4 h at room temperature. Then, the separated precipitate was washed with distilled water (2 × 20 mL) and ethanol (1 × 20 mL) and dried for 24 h at 50 °C. The inductively coupled plasma optical emission spectrometry (ICP-OES) and elemental analysis results allowed us to propose the formula: Eu_0.1_(NH_4_)_0.9_[Cu_3_(OH)(C_4_H_2_N_2_O_2_)_3_]·7H_2_O. Elemental analysis calcd (%): C 20.72, H 3.57, N 13.90; found: C 21.22, H 3.90, N 14.35. ICP-OES (wt.%): 26.27 ± 0.54 Cu, 3.33 ± 0.04 Eu. The ion-exchange process was also carried out for 2 h and 6 h in order to determine the effect of reaction time on the europium content (Table S1).

### 2.3. Stability test of Eu@Cu-MOF

To test the stability of Eu@Cu-MOF, 15 mg of the material was mixed with 15 mL of deionized water for 30 min, 1 h, 4 h, and 24 h. Then, the material was collected by centrifugation, dried for 24 h at 50 °C, and analyzed using X-ray powder diffraction. In addition, IR and Raman spectra were recorded for the powders after the studied time.

### 2.4. Detection of Ascorbic Acid

Luminescent responses of Eu@Cu-MOF toward an aqueous solution of small biological molecules were investigated. Firstly, aqueous solutions of glucose, leucine, arginine, glutamic acid, acetic acid, citric acid, formic acid, or ascorbic acid at a concentration of 1.00 × 10^−2^ M were prepared. Afterward, 1 mL of solution was mixed with a suspension of Eu@Cu-MOF (1 mg/mL in deionized water) and sonicated for 15 min. Then, the mixtures were used for fluorescent measurements. Experiments were also carried out for AA at a concentration range from 1.00 × 10^−2^ M down to 3.55 × 10^−4^ M. For the selected AA concentration equal to 7.10 × 10^−4^ M, the detection time of 5, 10, 15, and 30 min was also verified. Moreover, the filtrates after different treatment times were analyzed using ICP-OES to determine a possible release of the Eu and Cu ions. What is more, the dependence of the ion-exchange process on the europium content in the sample, and in consequence, on the detection limit of AA, was also investigated (Table S1, Figure S1).

### 2.5. Instrumentation

The content of the C, H, and N elements was determined by the CHNS Vario EL Cube Elementar. The structure of the powder samples was analyzed by X-ray diffraction (XRD) using an X’Pert PRO diffractometer (PANalytical) with a Cu Kα radiation (λ = 1.5406 Å). The specific surface area was determined based on the N_2_ sorption measurements. The N_2_ adsorption–desorption isotherms were obtained at 77 K using the Micromeritics 3Flex Surface Characterization Analyzer. Before isotherm acquisition, the materials were activated and outgassed (150 °C, 1.3 kPa) for 12 h. MicroActive software was used to determine: (i) the specific surface area according to the Brunauer–Emmett–Teller (BET) method; (ii) the pore size distribution using the BJH adsorption method (thickness curve: Halsey, BJH correction: standard, cumulative reports: larger, based on an isotherm analysis in the range 0.4 < p/p_0_ < 1); and (iii) total pore volume at a relative pressure (p/p_0_) = 0.9. A thermogravimetric (TG) analysis was conducted using the Setaram SETSYS TG-DTA 16/18 at a heating rate of 10 °C/min in flowing air. The mid-IR spectra were measured using the Nicolet iSTM50 FT-IR spectrometer. The spectra were recorded for the KBr pellets. The micro-Raman apparatus (inVia™ Renishaw) was used to register the Raman spectra with a 514 nm excitation line. The morphology and EDS mapping of the Eu@Cu-MOF were determined using a field emission scanning electron microscope (FE-SEM) FEI Nova NanoSEM 230 equipped with an energy dispersive X-ray spectrometer (EDAX Genesis XM4). The FLS980 spectrophotometer (Edinburgh Instruments) with a xenon lamp as an excitation source was used for photoluminescence measurements. The spectra were recorded at room temperature with the lamp and detector corrections. The chemical composition of Eu@Cu-MOF and the filtrates for the Eu and Cu content after treatment with AA were characterized using the iCAP™ 7400 ICP-OES Analyzer. Filtrates, after a defined mixing time, were acidified with 2 mL of concentrated HCl (35–38%). To quantify the content of Eu and Cu in Eu@Cu-MOF by ICP-OES, 1.0–1.2 mg of materials was treated with 1 mL of HCl (35–38%). After, the samples were diluted with distilled water up to 21 mL. ICP-OES measurements were performed in 3 replicates. X-ray photoelectron spectroscopy (XPS) was used for the surface chemical composition analysis. The non-monochromatized X-ray Mg Kα excitation source was used. All measurements were performed using an AES/XPS system EA10 (Leybold-Heraeus GmbH, Cologne, Germany). The overall resolution of the spectrometer during measurements was 0.96 eV as a full width at half-maximum (FWHM) of the Ag3d_5/2_ line. During measurements, the pressure was kept at a 10^−9^ mbar range. All acquired spectra were calibrated to adventitious carbon C1s at 285 eV. After subtraction of the linear-type background, the core-level spectra were decomposed into main components with mixed Gaussian–Lorentzian lines (70% G + 30% L for the majority of photopeaks) by a nonlinear least squares curve-fitting procedure using CasaXPS software. 

## 3. Results and Discussion

### 3.1. Synthesis and Characterization

**Figure 2:**
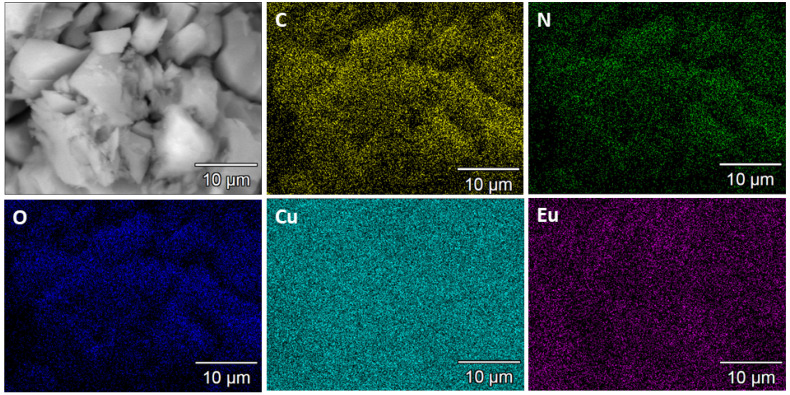
STEM-EDS mapping of C, N, O, Cu, and Eu for Eu@Cu-MOF.

The novel Eu@Cu-MOF material was obtained by exchanging an NH_4_^+^ cation for Eu^3+^ in a previously described unique anionic NH_4_[Cu_3_(μ_3_-OH)(μ_3_-4-carboxypyrazolato)_3_] [43], which is a 3D porous framework built up of trinuclear Cu_3_(μ_3_-OH) clusters connected to six others through μ_3_-4-carboxypyrazolato bridges [43]. The tetrahedral cages generated in this way host two extraframework NH_4_^+^ cations and the water of crystallization molecules. The new Eu[Cu_3_(μ_3_-OH)(μ_3_-4-carboxypyrazolato)_3_] system contained 3.33 wt.% of europium. The results of the ICP-OES and elemental analyses were indicative of Eu^3+^ ions giving rise to the exchange of 10% NH_4_ cations, resulting in the formula (Eu)_0.1_(NH_4_)_0.9_[Cu_3_(μ_3_-OH)(μ_3_-4-carboxypyrazolato)_3_]∙7H_2_O, for simplicity indicated as Eu@Cu-MOF. Based on the EDS maps of the analyzed area, a high dispersion of europium was found, which indicated that the ion-exchange process allowed the formation of a homogeneous material (Figure 2).

**Figure 3:**
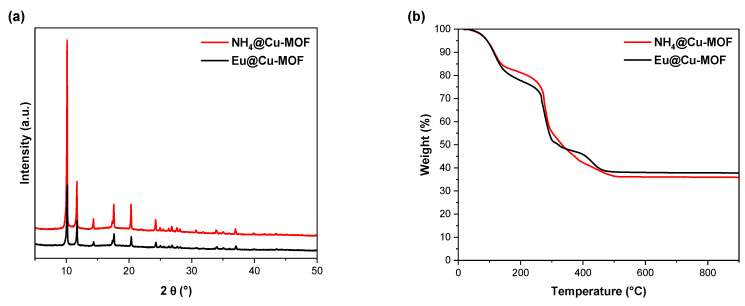

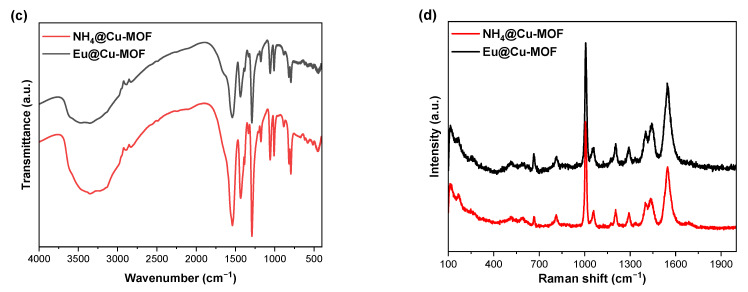
Characterization of Eu@Cu-MOF and NH_4_@Cu-MOF using (**a**) XRD, (**b**) TGA, (**c**) FTIR, and (**d**) Raman spectroscopy.

The properties of the derived material were investigated by different analytical techniques. In order to determine how the ion exchange process affected the structure and textural or thermal properties of Cu-MOF, a characterization was conducted for both NH_4_@Cu-MOF and Eu@Cu-MOF materials. The XRD patterns (Figure 3a) showed no significant difference between Eu@Cu-MOF and NH_4_@Cu-MOF, thus the structure remained stable even after the cation exchange. However, a slight decrease in the crystallinity could be observed after the process. The thermal stability of anionic MOF was evaluated by a thermogravimetric analysis. A high weight loss (in a similar range of 62–64%) could be noted for both systems (Figure 3b). According to the literature data, the weight loss in the first stage from 20 to 152 °C is indicative of sequential dehydration (−7H_2_O, calc./exp.: 18/18%). Further weight loss up to 450 °C is caused by the collapse of the skeleton [38]. 

The FTIR spectra (Figure 3c) show no additional bands in the molecular structure after the cation exchange process. The most intense band at 1290 cm^−1^ indicates the C–N stretching vibrations of the pyrazole ring [44]. Bands located at 1541 and 1436 cm^−1^ are attributed to the ν_asym_(COO^−^) and ν_sym_(COO^−^) stretching vibrations, respectively [45]. The value of the spectral split between the (COO^−^) modes, Δν_exp_, below 164 cm^–1^ (here 105 cm^−1^) usually indicate chelating or bridging carboxylate groups [46]. In fact, the crystal structure of Cu-MOF confirmed the chelate bidentate binding mode of carboxylates [43]. Moreover, the lack of the band at 1680–1740 cm^−1^, indicates that all carboxylic groups have been deprotonated [47].

A weak band at 445 cm^−1^ may be assigned to the stretching modes of Cu–O related to the oxygen of the carboxylate bridges [48]. A broad band covering the range of about 3000–3700 cm^−1^ is due to the O–H stretching vibrations of H_2_O.

A further spectroscopic characterization of the material was extended to Raman spectra (Figure 3d). Raman bands in the range from 110 to 550 cm^−1^ are visible due to the modes involving Cu^2+^ ions [48]. The most intense Raman band located at 1006 cm^−1^ may be assigned to the symmetric stretching mode of C=C [48]. The range from 1400 to 1600 cm^−1^ is usually attributed to the vibrational mode of carboxylate groups [48]. The band at 1446 cm^−1^ is attributed to the symmetric stretching of O–C=O, while the band at 1546 cm^−1^ may be assigned to the asymmetric vibration of O–C=O [48]. The presence of bonds recorded by Raman spectroscopy was also confirmed using the XPS method.

**Figure 4:**
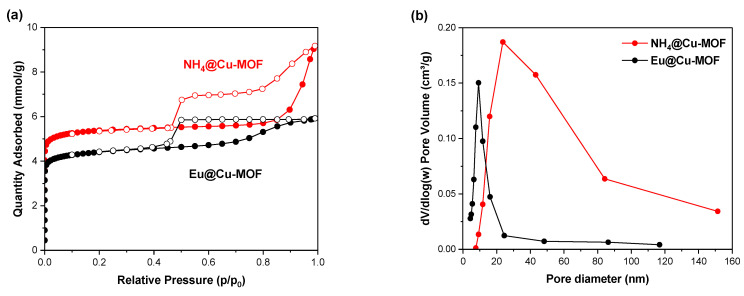
N_2_ adsorption–desorption (77 K) isotherms (**a**) and pore size distribution (**b**) of NH_4_@Cu-MOF and Eu@Cu-MOF.

Finally, an evaluation of the porosity and surface area was carried out. The N_2_ adsorption–desorption isotherms are shown in Figure 4a. The recorded isotherms were a combination of IUPAC’s types I and IV isotherms [49]. Therefore, the synthesized materials possessed micropores and mesopores [50]. Hysteresis loops provide information related to the distribution, shape, and connectivity of mesopores [50,51]. Both materials exhibited H2-type hysteresis, which according to the IUPAC classified hysteresis loops is associated with ink-bottle-shaped pores with poor connectivity and uneven pore structure [49]. It can be noted that the ion-exchange process modulated the porosity of the MOF. On passing from NH_4_^+^ to Eu^3+^, the BET surface area decreased from 476 to 387 m^2^/g, while pore volume reduced slightly from 0.22 to 0.20 cm^3^/g. This may be a result of the partial occupation of pores by Eu^3+^. Both materials exhibited a unimodal pore size distribution (Figure 4b); however, it became narrower after the cation exchange process, probably due to the nature of Eu^3+^ ions.

### 3.2. Luminescent Properties of Eu@Cu-MOF

**Figure 5:**
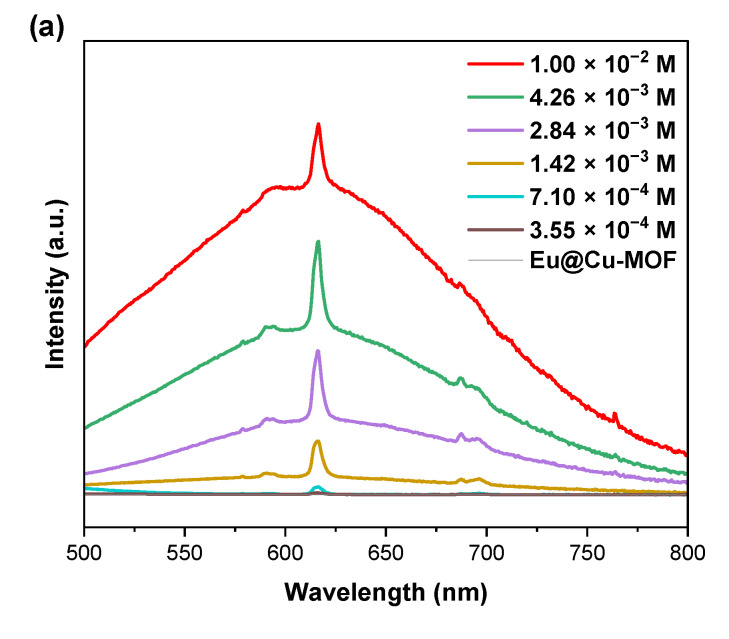

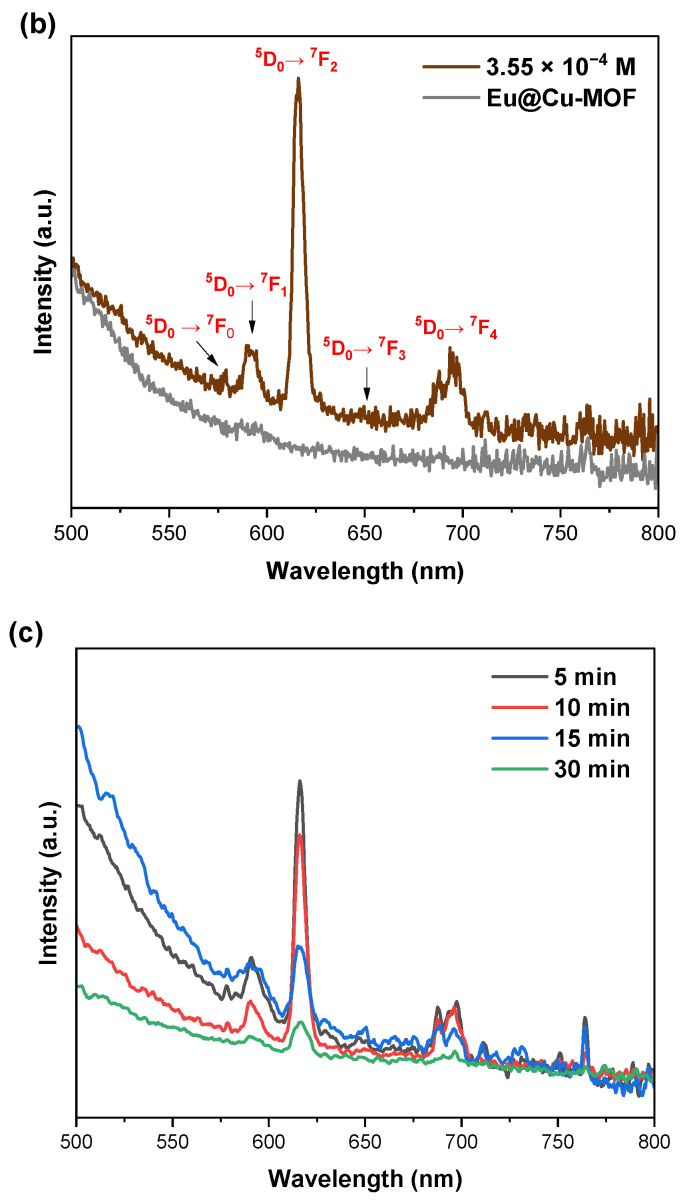
Emission spectra of Eu@Cu-MOF: (**a**,**b**) for different concentrations of AA (after treatment for 15 min); (**c**) for different times of treatment with AA (C = 7.10 × 10^−4^ M, λ_exc_ = 330 nm).

The luminescent properties of Eu@Cu-MOF were investigated in a water suspension at room temperature. Eu@Cu-MOF showed no emission in water under a 330 nm excitation (Figure S2). The characteristic emission bands originating from europium ions (arising from the ^5^D_0_ → ^7^F_0–4_ transitions) could be observed after the addition of AA (Figure 5). The origin of this effect is explained further on.

First, in order to study the sensitivity of the system to AA detection, various concentrations of the acid were added into the Eu@Cu-MOF suspension, followed by a 15 min ultrasonic treatment. All emission spectra of Eu@Cu-MOF in the presence of different concentrations of AA (Figure 5a,b) showed five emission bands at 579 nm, 593 nm, 617 nm, 651 nm, and 695 nm, which were assigned to the transition peaks of ^5^D_0_ → ^7^F_0_, ^5^D_0_ → ^7^F_1_, ^5^D_0_ → ^7^F_2_, ^5^D_0_ → ^7^F_3_, and ^5^D_0_ → ^7^F_4_, respectively. The spectroscopic properties of the europium ions often allow to obtain crucial information about the symmetry of the surrounding Eu^3+^ in the material matrix. The relative intensities of the bands and the presence of the ^5^D_0_→^7^F_0_ transition could indicate that europium ions were located in the low-symmetry environment [52].

It may be noted that the emission intensity of europium ions increased with the increase of AA concentration up to 2.84 × 10^−4^ M. For higher concentrations, the intensity remained at the same level (Figure 5a). The detection limit was 3.55 × 10^−4^ M as a turn-on probe (for lower concentrations, no emission bands were registered). To determine the shortest time needed for the detection of AA through Eu@Cu-MOF, experiments were carried out for 5, 10, 15, and 30 min of interaction between the components for a selected concentration of AA equal to 7.10 × 10^−4^ M. Importantly, after only 5 min of treatment, AA was successfully detected (Figure 5c). What is more, the longer the treatment time with AA, the lower the intensity of luminescence observed. It may be a consequence of the structural changes in the metal–organic framework or the release of ions into the solvent, as shown hereafter. It is worth to underline that the obtained response was quick compared to other systems known in the literature. For the MOF [{(H_3_O)[Eu(SBDB)(H_2_O)_2_]}*_n_*] (where H_4_SBDB = 1,5-disulfo-benzene-2,4-dicarboxylic acid) proposed by Yuan et al. [38], 30 min were needed to observe emission bands of europium. Among MOF-based sensors, those based on other ions, such as cadmium [53] or iron [54], are also known to require longer times to detect AA, such as 10 and 15 min, respectively.

**Figure 6:**
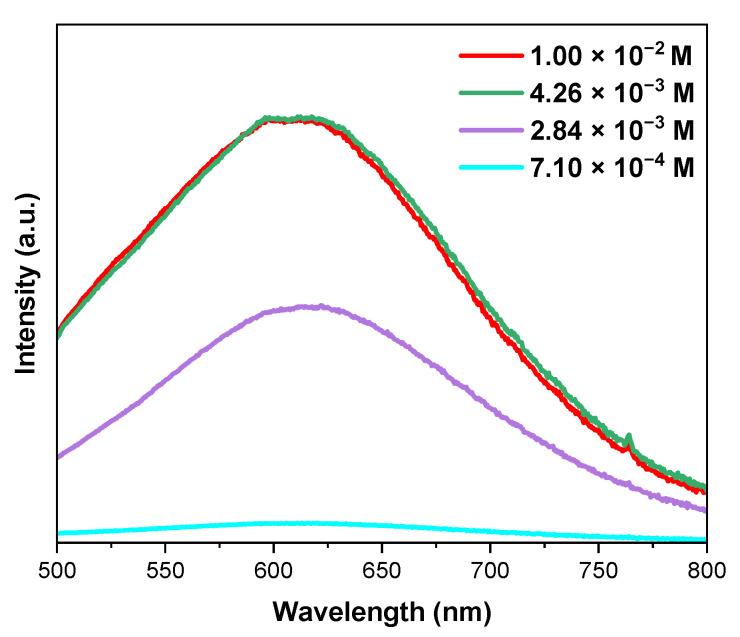
Emission spectra of NH_4_@Cu-MOF for different concentrations of AA (after treatment for 15 min, λ_exc_ = 330 nm).

Above a concentration of 7.10 × 10^−2^ M of AA, except for bands originating from europium(III) transitions, an intense broad band with a maximum at 616 nm was observed. In order to determine the origin of that band, a treatment with AA at a concentration from 7.10 × 10^−4^ M to 1.00 × 10^−2^ M was performed for NH_4_@Cu-MOF (Figure 6). The emission spectra for the structure without Eu^3+^ revealed the same broadband emission, which could be connected to the reduction of Cu(II) to Cu(I) by AA [44]. It can be noted that with an increasing concentration of the acid, the emission intensity was higher, thus a greater amount of copper(II) was probably reduced. Similarly, a broad emission with a maximum at 616 nm was observed for the Cu(I) pyrazolate complex [44,55].

**Figure 7:**
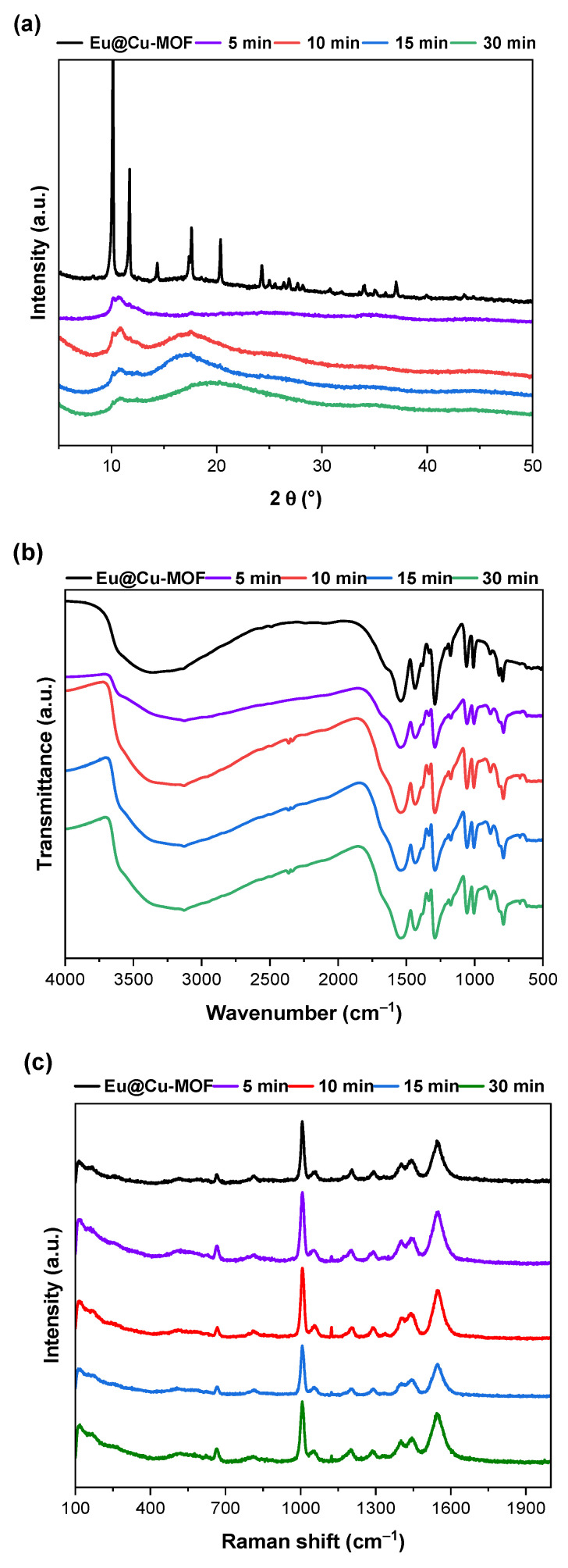
Determination of stability of Eu@Cu-MOF after treatment with AA at a concentration of 7.10 × 10^−4^ M using (**a**) XRD patterns, (**b**) IR, and (**c**) Raman spectroscopies.

To evaluate the ·×stability of the Eu@Cu-MOF system, samples after treatment with AA were analyzed using XRD, IR, and Raman spectroscopies (Figure 7). The XRD patterns showed that after treatment with AA, the crystallinity of the metal–organic framework decreased significantly (Figure 7a). To determine whether it was affected by AA, stability tests were conducted in pure deionized water. NH_4_@Cu-MOF has been reported as an example of a water-stable system (up to 353 K for 24 h) [43]. However, after the cation exchange process, the crystallinity of Eu@Cu-MOF after water exposure decreased significantly, as evidenced by XRD (Figure S3a). On the other hand, no significant changes were observed in the IR and Raman spectra of Eu@Cu-MOF (Figure S3b,c) after treatment in deionized water. Similarly, the loss of crystal structure was observed after the reaction with AA in water, with no noteworthy changes in the IR and Raman spectra (Figure 7b,c). Additionally, the ICP-OES analysis of filtrates after various times of AA testing revealed that the concentrations of both europium and copper ions increased with time, which would indicate their release from the framework during the treatment with AA (Figure S4).

Eventually, the interaction of Eu@Cu-MOF with other biological reducing agents (at 10 mM concentration) was investigated in the studies. Ascorbic acid was the only one among the tested compounds that led to the occurrence of the Eu^3+^ emission. No photoluminescence observed in the case of leucine, glutamic acid, arginine, glucose, formic acid, citric acid, and acetic acid (Figure S5) proved the high selectivity of Eu@Cu-MOF towards AA. In the case of the system proposed by Zheng et al. [37], other species, such as cysteine, homocysteine, PO_4_^3−^, CO_3_^2−^, Fe^3+^, Zn^2+^, or Ca^2+^, also caused a detectable emission, but the fluorescence intensity was remarkably higher for AA.

Most of the optical sensors using fluorescence signal show emission quenching and “turn-on” responses (an emergence of luminescence after adding an analyte) are relatively rare [44]. To thoroughly understand the observed effect, a possible ongoing mechanism of interactions needs to be determined.

In the MOF system, the sharp-band emission under UV excitation may occur due to the antenna effect among the ligand and Eu^3+^ ions. However, copper(II) ions present in the framework structure effectively suppress this photoluminescence [56,57,58], which is not observed for Eu@Cu-MOF. When added to the medium, the ascorbic acid must interact with copper(II) ions, which significantly weaken the quenching effect of Cu^2+^, and thus the emission of Eu^3+^ can be observed. The most probable mechanism is based on the redox reaction between the sensing system and the analyte. In the presence of AA, Cu^2+^ may transform into Cu^+^ ions [59]. The copper reduction process is accompanied by the oxidation of ascorbic acid which generates dehydroascorbic acid (C_6_H_6_O_6_, DHA). The enediol group of AA may be oxidized to two ketone groups leading to the DHA having three ketone groups (one from AA and two from oxidation) [14]. Cu^2+^ has a tendency to gain electrons due to its unsaturated electronic state. It may also promote the interaction between Cu^2+^ ions and carboxylic oxygen from the ligand through the cooperative effect and formation of O–Cu–O bonds. It may further increase the ligand–copper(II) charge transfer and simultaneously reduce the ligand–europium(III) charge transfer, which in consequence does not favor the emission of Eu^3+^ ions [37]. One may conclude that the reduction of Cu^2+^ to Cu^+^ (after AA treatment) is responsible for the ongoing emission of Eu^3+^ ions since the O–Cu–O are created less effectively, and simultaneously, the energy transfers are disturbed (light emissions related to ligand-to-metal (Eu^3+^) and metal-to-ligand (Cu^+^) charge transfers are observed).

**Figure 8:**
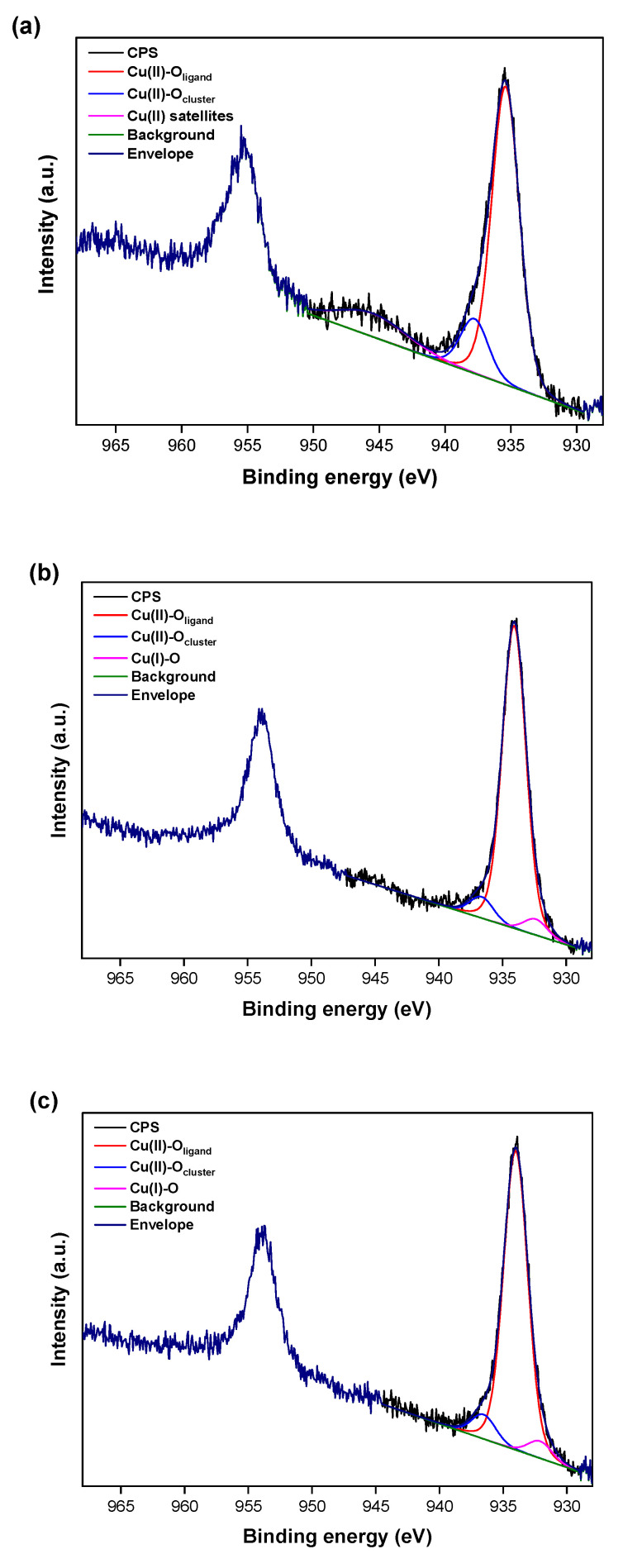
The high-resolution XPS spectra for Cu 2p of Eu@Cu-MOF before (**a**) and after 5 min (**b**) and 30 min (**c**) of treatment with AA at a concentration equal to 1.00 × 10^−2^ M.

This proposed mechanism could be confirmed by the XPS studies, which revealed the Cu(I) species in a region of Cu 2p_3/2_ after treatment with AA (Figure 8). Two peaks at 936.6 eV and 934.1 eV were characteristic for Cu(II)–O species [58], while the peak at 932.3 eV assigned to Cu(I) state [60,61] was noted in the spectra of Eu@Cu-MOF after 5 min (Figure 8b) and 30 min (Figure 8c) of the reaction with AA. After ascorbic acid treatment, the percentages of individual components also changed (Table S2) and the amount of Cu(I) species increased from 4.7% to 5.03% during 30 min of AA treatment.

The optical responses of MOFs to analytes have different backgrounds and can be divided into mechanisms based on the collapse of frameworks, photoelectron transfer, resonant energy transfer, or competitive absorption [31]. In the case of Eu@Cu-MOF, the collapse of the crystal structure was observed both in the case of treatment with pure water and an aqueous solution of AA (Figure S3a and 7a, respectively). Water does not cause Eu^3+^ luminescence so the structure modification was not responsible for the observed effect (moreover, in the case of framework collapse, the decrease of luminescence would be rather expected). Therefore, an interaction between the analyte (in a direct or indirect way) and the complex (ligand or metal ion) was expected.

Similar systems used for AA detection worked in a slightly different way than the one described in this report. The luminescence of Eu^3+^ ions in a MOF structure based on 1,5-disulfobenzene-2,4-dicarboxylic acid was significantly quenched by AA, which bonded to the ligand and reduced the ligand-to-europium(III) charge transfer [38]. The reported detection limit was low (50 µM), but other molecules also had a certain quenching effect on the complex. Zheng et al. [37] presented a turn-on sensing system of a Eu(III)–Cu(II) heterometallic–organic framework, namely [EuCu(pydc)_2_(ox)_0.5_(H_2_O)_3_·1.5H_2_O]_2*n*_ (H_2_pydc = 2,3-pyridinedicarboxylic acid and ox = oxalic acid), with an estimated detection limit of 55 nM. However, its reaction was obtained only after the addition of H_2_O_2_. The studies showed that in the presence of the complex, hydrogen peroxide was decomposed into hydroxyl radicals which formed oxidized AA. As claimed by the authors, the latter molecules were responsible for an efficient Eu^3+^ fluorescence enhancement [37]. Eventually, another mechanism was reported by Yue et al. [14], who used a mixed valence state cerium-MOF with 1,1′:4′,1′′-terphenyl-2′,4,4′′,5′-tetracarboxyliate ligand. After adding AA to the complex suspension, the intensity of the luminescent signal increased. In this case, AA caused a reduction of Ce^4+^ to Ce^3+^. Generated dehydroascorbic acid blocked the photoinduced electron transfer process from the ligand to the metal ion, and therefore the fluorescence of the ligand was enhanced. Looking at those results, it may be concluded that the proposed Eu@Cu-MOF could be an interesting alternative for the ascorbic acid detection and the design of other sensing systems.

## 4. Conclusions

Herein, we demonstrated a simple way to gain photoluminescent properties for an anionic metal–organic framework using the cation-exchange process of the extraframework NH_4_^+^ cations of NH_4_[Cu_3_(μ_3_-OH)(μ_3_-4-carboxypyrazolato)_3_] (NH_4_@Cu-MOF) to Eu^3+^ leading to the Eu@Cu-MOF material. The proposed system enabled the detection of ascorbic acid, an important biomolecule, the deficiency of which is associated with the symptoms of many diseases. Eu@Cu-MOF revealed a selective “turn-on” fluorescent response in the presence of AA in aqueous media for concentrations above 3.55 × 10^−4^ M. The shortest tested detection time reached 5 min, while a longer interaction of the compound with water molecules caused a disorder in the MOF structure. A mechanism of interaction between the sensing system and the analyte based on a Cu^2+^ reduction was proposed.

## Data Availability

Not applicable.

## Supplementary Materials

The following supporting information can be downloaded at: https://www.mdpi.com/article/10.3390/nano12244480/s1, Figure S1: Emission spectra of Eu@Cu-MOF after cation exchange for 2 h (a) and 6 h (b) for different concentrations of AA (after treatment for 15 min); Table S1:Content of Eu and Cu (wt.%) in NH_4_[Cu_3_(µ_3_-OH)(µ_3_-4-carboxypyrazolato)_3_] after different times of cation-exchange process based on ICP-OES; Figure S2: Excitation spectra of Eu@Cu-MOF (λ_em_ = 616.5 nm); Figure S3: Determination of stability of Eu@Cu-MOF in deionized water using (a) XRD, (b) IR, and (c) Raman spectroscopy; Figure S4: Changes in the concentration of europium and copper in the solution after treatment of Eu@Cu-MOF with ascorbic acid; Figure S5: Emission spectra for Eu@Cu-MOF for different aqueous solutions of biological molecules and carboxylic acids at a concentration of 1.00 × 10^−2^ M; Table S2: Relative composition (%) of the Cu species.
