# Supplementary material for: Cation-Exchange in Metal-Organic Framework as a Strategy to Obtain New Material for Ascorbic Acid Detection
Source: Nanomaterials (Basel). 2022 Dec 18;12(24):4480. doi: 10.3390/nano12244480 (PMC9786631; doi:10.3390/nano12244480)
Supplement: Supplementary file 1 [file nanomaterials-12-04480-s001.zip › nanomaterials-2057652-supplementary.pdf]

Cation-Exchange in Metal–Organic Framework as a Strategy to Obtain New Material for Ascorbic Acid Detection

Weronika Bodylska [1,*], Marzena Fandzloch [1], Rafał Szukiewicz [2] and Anna Lukowiak [1]

[1] Institute of Low Temperature and Structure Research, Polish Academy of Sciences, Okólna 2, 50-422 Wrocław, Poland

[2] Faculty of Physics and Astronomy, University of Wrocław, pl. M. Borna 9, 50-204 Wrocław, Poland

*Corresponding author: w.bodylska@intibs.pl

Table of Contents

Table S1 ……………………..………………………………………………………….. 3

Figure S1 ……………………..…………………………………………………………. 3

Figure S2 ……………………..…………………………………………………………. 4

Figure S3 ……………………..…………………………………………………………. 5

Figure S4 ……………………..…………………………………………………………. 6

Figure S5 ……………………..…………………………………………………………. 6

Table S2 ……………………..…………………………………………………………. 7

Table S1. Content of Eu and Cu (wt.%) in $NH_4[Cu_3(\mu_3\text{-OH})(\mu_3\text{-4-carboxypyrazolato})_3]$ after different times of cation-exchange process based on ICP-OES.

Sample	Element content (wt.%)
2 h of ion-exchange process	Cu: 26.16 ± 0.83<br>Eu: 2.66 ± 0.18
4 h of ion-exchange process	Cu: 26.27 ± 0.54<br>Eu: 3.33 ± 0.04
6 h of ion-exchange process	Cu: 26.91 ± 0.41<br>Eu: 4.30 ± 0.05

As far as the cation exchange time is concerned, shorter (2 h) and longer (6 h) time was also tested. Eu@Cu-MOF obtained after 2 h of the cation-exchange process showed a characteristic emission band of europium at 617 nm and a broad copper-originating band for the concentration of AA of 2.84 $\times 10^{-3}$ M (Figure S1a) but not for lower concentrations. For the initially proposed time of the ion-exchange process (4 h) the detection limit of AA was much lower (set up at 3.55 $\times$ 10^{-4} M). The longer time, up to 6 h of the process, did not change the detection limit of AA (Figure S1b). As presented in Table S1, the europium content in the sample after 2 h of the ion-exchange process was estimated to be 2.66 wt.%, which is lower than in the system after 4 h of reaction (3.33 wt.%). Therefore, it may explain the lower sensitivity of the system after 2 h-exchange. On the other hand, europium content in the sample after 6 h of the ion-exchange process was higher than for 4 h (4.30 wt.%); however, it did not change the detection limit of the proposed system.

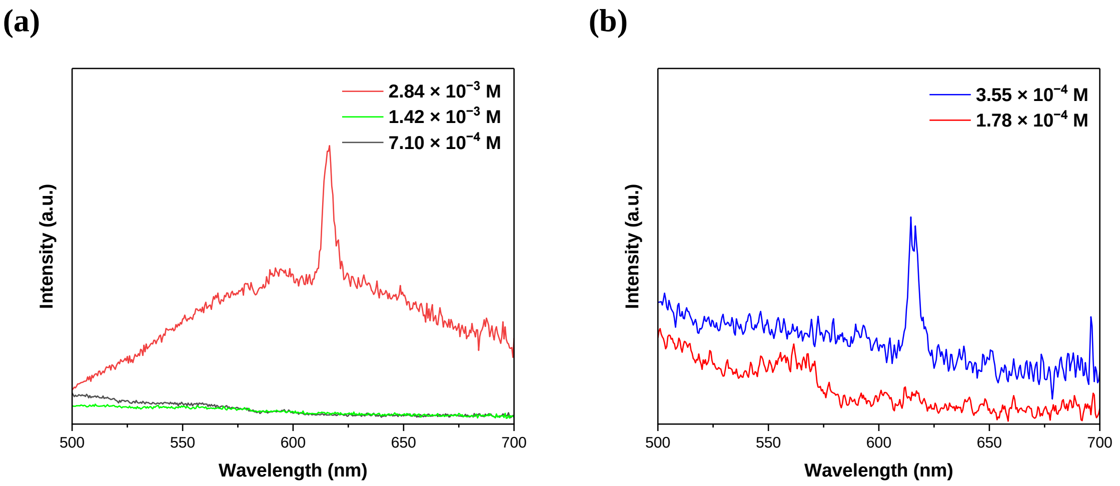

Figure S1. Emission spectra of Eu@Cu-MOF after cation exchange for 2 h (a) and 6 h (b) for different concentrations of AA (after treatment for 15 min).

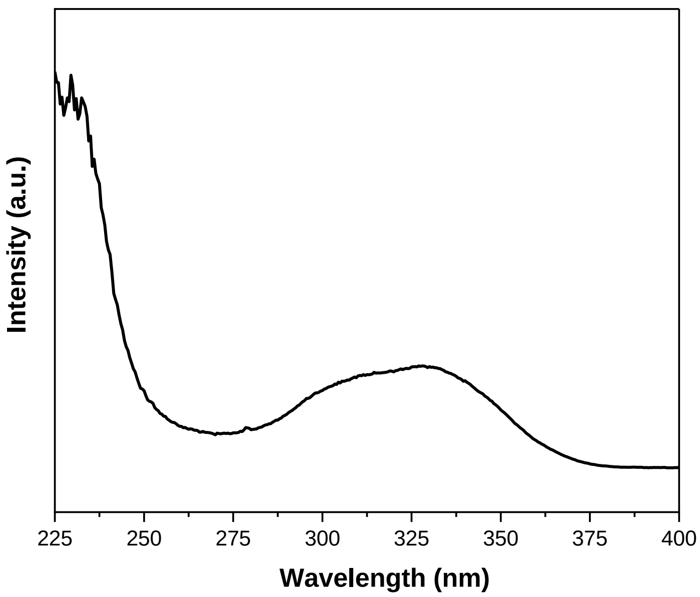

Figure S2. Excitation spectra of Eu@Cu-MOF (λ_{em} = 616.5 nm).

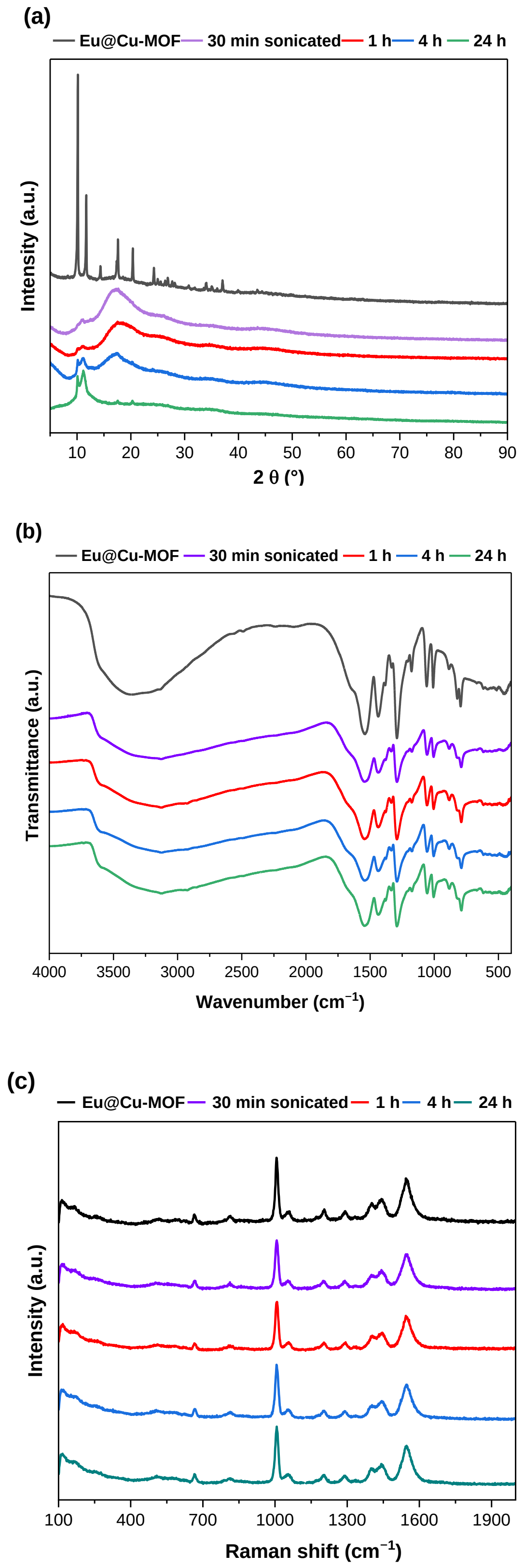

Figure S3. Determination of stability of Eu@Cu-MOF in deionized water using (a) XRD, (b) IR, and (c) Raman spectroscopy.

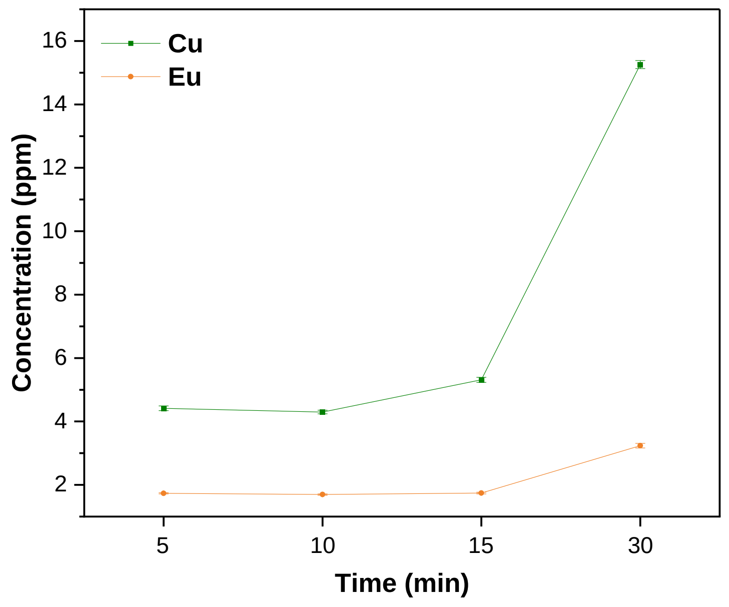

Figure S4. Changes in the concentration of europium and copper in the solution after treatment of Eu@Cu-MOF with ascorbic acid.

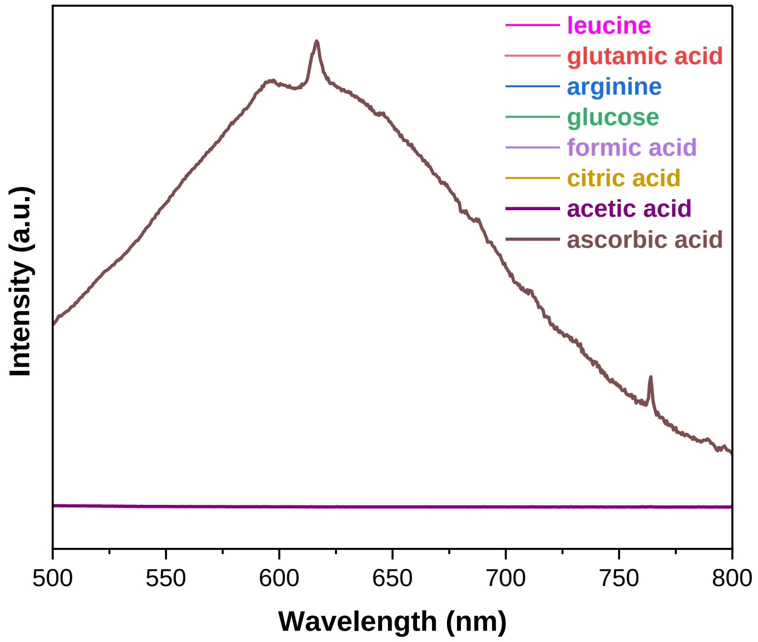

Figure S5. Emission spectra for Eu@Cu-MOF for different aqueous solutions of biological molecules and carboxylic acids at a concentration of 1.00×10^{-2} M.

Table S2. Relative composition (%) of the Cu species.

	Cu(II)–O_{ligand}	Cu(II)–$O_{cluster}$	Cu(I)
Eu@Cu-MOF	84.49	15.51	0
5 min	89.03	6.27	4.7
30 min	87.8	7.17	5.03
